# Demographic Model of the Swiss Cattle Population for the Years 2009-2011 Stratified by Gender, Age and Production Type

**DOI:** 10.1371/journal.pone.0109329

**Published:** 2014-10-13

**Authors:** Sara Schärrer, Patrick Presi, Jan Hattendorf, Nakul Chitnis, Martin Reist, Jakob Zinsstag

**Affiliations:** 1 Veterinary Public Health Institute/University of Berne, Berne, Switzerland; 2 Swiss Tropical and Public Health Institute/University of Basel, Basel, Switzerland; 3 Fogarty International Center, National Institutes of Health, Bethesda, Maryland, United States of America; 4 Federal Food Safety and Veterinary Office, Bern, Switzerland; ETH Zurich, Switzerland

## Abstract

Demographic composition and dynamics of animal and human populations are important determinants for the transmission dynamics of infectious disease and for the effect of infectious disease or environmental disasters on productivity. In many circumstances, demographic data are not available or of poor quality. Since 1999 Switzerland has been recording cattle movements, births, deaths and slaughter in an animal movement database (AMD). The data present in the AMD offers the opportunity for analysing and understanding the dynamic of the Swiss cattle population. A dynamic population model can serve as a building block for future disease transmission models and help policy makers in developing strategies regarding animal health, animal welfare, livestock management and productivity. The Swiss cattle population was therefore modelled using a system of ordinary differential equations. The model was stratified by production type (dairy or beef), age and gender (male and female calves: 0–1 year, heifers and young bulls: 1–2 years, cows and bulls: older than 2 years). The simulation of the Swiss cattle population reflects the observed pattern accurately. Parameters were optimized on the basis of the goodness-of-fit (using the Powell algorithm). The fitted rates were compared with calculated rates from the AMD and differed only marginally. This gives confidence in the fitted rates of parameters that are not directly deductible from the AMD (e.g. the proportion of calves that are moved from the dairy system to fattening plants).

## Introduction

Switzerland has been collecting data about cattle including date of birth, date of slaughter, date of death (other than slaughter for consumption) and information regarding movements on a mandatory basis since 1999. The purpose of a national database of animal movements was originally to restore consumer trust during the BSE crisis by assuring traceability and therefore a better food safety of beef products and to provide a tool for epizootic disease surveillance and control [1], [2]. The AMD contains detailed and complete datasets about the Swiss cattle population for several years offering the opportunity to get an insight into the population dynamics. Understanding the demographic of the livestock population in turn provides accurate parameters needed to develop models of disease transmission and helps policy makers in developing strategies regarding animal health, animal welfare and livestock management [3].

Early detection of disease, monitoring of present agents and substantiation of freedom from disease are described as key tasks of modern public veterinary services in order to allow international trade with agricultural goods and to document a good sanitary status of domestic livestock [4]–[6].

To monitor the health status of the cattle population, the Swiss veterinary authorities invest substantial resources in yearly surveillance programmes that have to meet international standards. One way to maintain the standards while reducing the costs is the application of risk based targeted approaches (e.g. [7]). Other approaches comprise logistical improvements such as better exploiting infrastructures where already a lot of potential information carriers are available e.g. slaughterhouse or milk quality testing laboratories [8]. With the implementation of bulk milk testing in 2010 [9], [10] the production type became an important criterion for shaping the sampling strategy of national surveillance programs. As beef and fattening cattle, correspond to one third of the population, they have to be handled separately. The two production types (dairy and beef) do not only differ with respect to purpose but also with respect to management practices. The resulting differences in age distribution and slaughter rates in the two sub populations are of interest for the planning of stratified surveillance programmes to assure the representativeness of the sample (e.g. for sampling at the slaughterhouse level).

The objective of this study was therefore to create an AMD data driven demographic model that simulates the age and gender specific dynamics of the Swiss cattle population according to the production type. The derived rates describing population dynamics can be used for livestock development planning and associated economic analyses, as a backbone for disease transmission models or for the design of cost-effective disease control and monitoring programmes.

Here we present the first dynamic demographic model of the Swiss cattle population. It is based on over 30 million data points collected in the Swiss animal movement database (AMD) between 2009 and 2011.

## Materials and Methods

### 2.1 The Swiss cattle population

**Figure 1:**
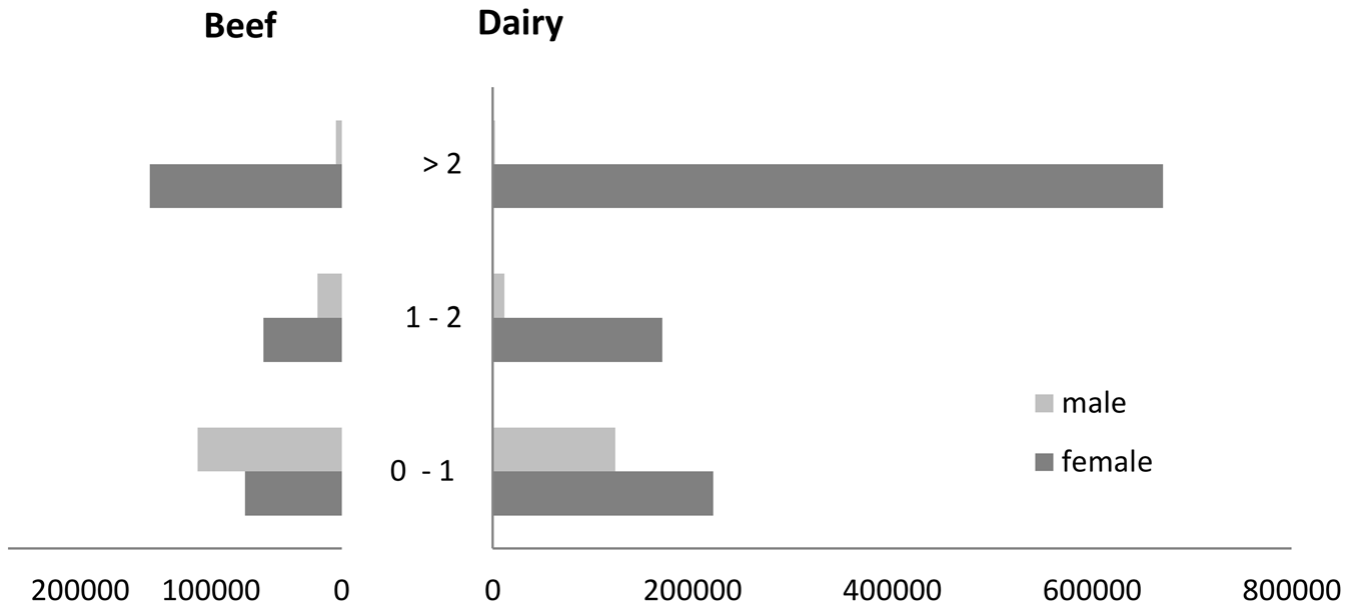
Demographic of the Swiss cattle population per age class and sex in number of animals.

The major livestock species in Switzerland is cattle. Although the number of farms decreases, for the years 2009–2011 the number of cattle in Switzerland is stable at roughly 1.6 million animals (table 1). Two thirds of the Swiss cattle industry is dedicated to dairy production. As a consequence, adult dairy cows (older than two years) make the largest demographic segment (figure 1). The average lifespan of a dairy cow in Switzerland is 6.2 years and the average number of calves in a lifetime is 3.7. The oldest cow that died between 2009 and 2011 was 25 years old.

**Table 1:** The Swiss cattle population 2009–2011.

Year	No of farms	No of cattle (January 1th)	No of dairy cows (January 1th)	No of slaughtered animals	No of births
2009	42‘966	1‘608‘062	675‘285	647‘715	721‘810
2010	42‘233	1‘610‘277	671‘874	648‘313	719‘004
2011	41‘465	1‘612‘230	676‘253	653‘754	718‘697

Numbers are extracted from the Swiss animal movement database (AMD).

Due to subsidies for ecological and behaviourally sound husbandry and strict animal protection legislation, small holdings with less than hundred animals are still the most common farm type. Over the summer month (May–October) one fourth of the livestock is moved to alpine pastures.

### 2.2 Data management

The Swiss animal movement database (AMD) contains information on farm level (e.g. location, production type), animal level (e.g. birthdate, gender, and breed), movement records (date, movement type) and stays (i.e. for every animal the start and end date of a stay on any holding is recorded). The data used for the models was an extract from the AMD, containing all recorded movements (25.5 million entries) and stays (15.8 million entries) from January 1999 until January 2012.

Birthdate, date of death (slaughter or natural) and gender are recorded on individual animal level, while the production type is available on farm level. The production type for each animal was consequently determined by the farm it stayed on at the given time step. Calf mortality consisted of notified stillbirths and mortality. As stays on alpine pastures are recorded only since 2008 and the quality of those recordings improved notably in 2009, only data from 2009 to 2011 was used for fitting of the population model.

### 2.3 The model

**Figure 2:**
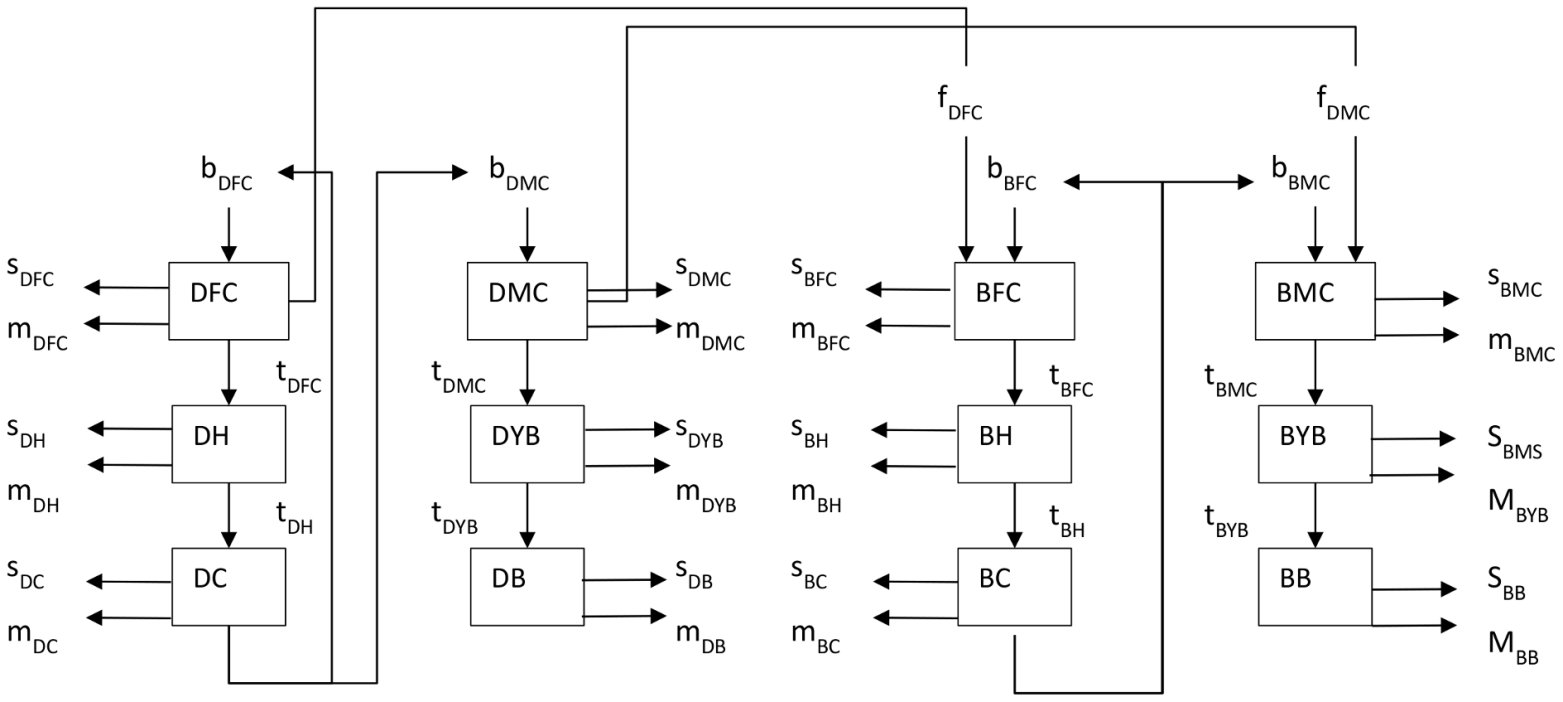
Schematic representation of the Vensim model. Arrows represent flows of animals into or out of a compound, boxes represents numbers of animals at a given time point in a category. s: slaughter; m: mortality; b: birth; tr: transition; f: fattening; D: dairy; B: beef; F: female; M: male; X: calves; Y: subadults; Z: adults.

The Swiss cattle population was simulated using a system dynamic software [11]. The model is composed of a series of coupled difference equations. Compartments were defined by production type (dairy or beef), age class and gender. Calves were defined as animal being less than one year old, heifer and young bulls as one to two years old and cows and bulls as older than two years. We assumed that cows calve for the first time at the age of two and therefore the category “heifer” doesn't contribute to births. The beef and dairy system are connected through the transfer of calves from dairy farms to fattening plants, which is represented in the model as “fattening”. The model is represented in figure 2.

The dynamic of the cattle population is simulated by month as time unit. Equations (1)–(12) show the number of animals per compartment (for parameter notation see table 2 and 3).

**Table 2:** Nomenclature for subscripts in Equations 1–12.

	Description	type
X	Calves	age class
Y	Subadults	age class
Z	Adults	age class
D	Dairy	production type
B	Beef	production type
F	Female	gender
M	Male	gender

**Table 3:** Compartments and parameters in Equations 1–12.

	Description	Unit
X	No of calves	Animals
Y	No of subadults	Animals
Z	No of adults	Animals
s	slaughter rate	month^−1^
m	mortality rate	month^−1^
b	birth rate	month^−1^
tr	transition rate	month^−1^
f	fattening rate	month^−1^
μ	Average	month^−1^
a	Amplitude	month^−1^
ω	Frequency	month^−1^
φ	Phase	Dimensionless

To represent the seasonal fluctuations in the number of births and death calves, we used a sinusodial-function with amplitude (a), phase (φ) and average (μ) as parameters to fit (equations (13)–(20)). The frequency (ω) was set to 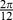.



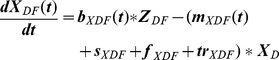
(1)




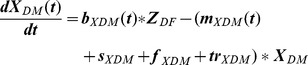
(2)




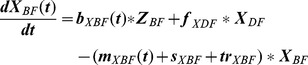
(3)




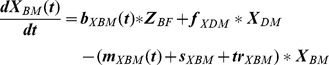
(4)




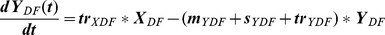
(5)




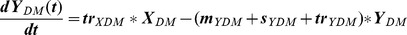
(6)




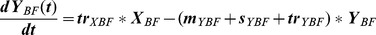
(7)




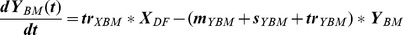
(8)




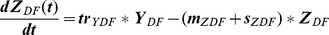
(9)




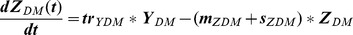
(10)




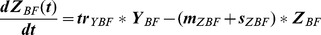
(11)




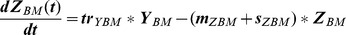
(12)




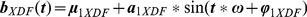
(13)




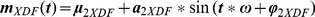
(14)




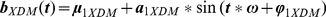
(15)




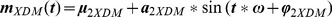
(16)




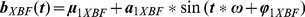
(17)




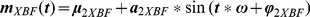
(18)




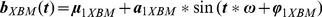
(19)




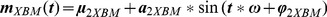
(20)


#### 2.3.1 Model fitting

The number of living animals was extracted at the beginning of each month, number of birth, slaughter and death from the AMD per month, age class, production type and gender from January 2009 to December 2011. This data-set served to optimize the model parameters on the basis of the goodness-of-fit of the nonlinear maximum-likelihood optimization using the Powell algorithm [12]. Parameters were fitted stepwise, adding a variable at every step to the payoff values, using the outcome rates from the previous step as initial search point (maximum and minimum values set to +/−10%).

#### 2.3.2 Comparison of calculated and fitted rates

Birth, slaughter and mortality rates were calculated from the AMD data and compared to the fitted values from the model. Average birth rates were calculated as number of calves per month and category divided by the number of cows on the first of the months of the according production type and averaged over the 3 years period. Mortality and slaughter rates were calculated as number of death or slaughtered animals per month divided by the number of animals of the same age category and production type on the first of the month and averaged over the 3 years period. Model and empirical estimates were correlated in R [13].

#### 2.3.3 Sensitivity analysis

The model was rebuilt with the statistical software R. To assess the sensitivity of the model, each parameter was varied separately using a range from −10% to +10% of the fitted value from the Vensim model (baseline), divided in 100 steps. For each value, the resulting absolute change in total numbers of animals compared to the baseline was represented graphically (Figures S1–S10, supplementary material).

## Results

In table 4 the fitted parameter values from the demographic model are shown. The model allowed the calculation of parameters that are not directly deductible from the AMD (transition rates and fattening rates).

**Table 4:** Monthly population parameters for the Swiss cattle population.

	Dairy				Beef		
			Month^−1^	95%-CI		Month^−1^	95%-CI
slaughter rates	Female calf	**s**_XDF_	0.0197	[0.0192, 0.0201]	**s**_XBF_	0.0396	[0.0389, 0.0403]
	Heifer	**s**_YDF_	0.0065	[0.0062, 0.0069]	**s**_YBF_	0.0261	[0.0253, 0.0269]
	Cow	**s**_ZDF_	0.0190	[0.0189, 0.0191]	**s**_ZBF_	0.0233	[0.0231, 0.0235]
	Male calf	**s**_XDM_	0.1123	[0.1103, 0.1144]	**s**_XBM_	0.0638	[0.0631, 0.0645]
	Young bull	**s**_YDM_	0.1702	[0.1658, 0.1748]	**s**_YBM_	0.2834	[0.2768, 0.2902]
	Bull	**s**_ZDM_	0.1113	[0.1072, 0.1156]	**s**_ZBM_	0.0606	[0.0590, 0.0623]
mortality rates	Female calf	**μ**_2XDF_	0.0094	[0.0089, 0.0098]	**μ**_2XBF_	0.0059	[0.0055, 0.0062]
	Heifer	**m**_YDF_	0.0007	[0.0006, 0.0007]	**m**_YBF_	0.0008	[0.0007, 0.0009]
	Cow	**m**_ZDF_	0.0013	[0.0012, 0.0013]	**m**_ZBF_	0.0013	[0.0013, 0.0014]
	Male calf	**μ**_2XDM_	0.0255	[0.0241, 0.0269]	**μ**_2XBM_	0.0074	[0.0071, 0.0078]
	Young bull	**m**_YDM_	0.0017	[0.0015, 0.0019]	**m**_YBM_	0.0017	[0.0015, 0.0019]
	Bull	**m**_ZDM_	0.0022	[0.0016, 0.0028]	**m**_ZBM_	0.0026	[0.0021, 0.0031]
transition rates	Female calf	**tr**_XDF_	0.0684	[0.0678, 0.0689]	**tr**_XBF_	0.0718	[0.0710, 0.0725]
	Heifer	**tr**_YDF_	0.0804	[0.0797, 0.0812]	**tr**_YBF_	0.0615	[0.0607, 0.0624]
	Male calf	**tr**_XDM_	0.0207	[0.0203, 0.0212]	**tr**_XBM_	0.0511	[0.0505, 0.0518]
	Young bull	**tr**_YDM_	0.0234	[0.0226, 0.0243]	**tr**_YBM_	0.0161	[0.0157, 0.0165]
fattening rates	Female calf	**f**_XDF_	0.0172	[0.0170, 0.0175]			
	Male calf	**f**_XDM_	0.0731	[0.0722, 0.0740]			
birth rates	Female calf	μ_1XDF_	0.0374	[0.0373, 0.0376]	**μ**_1XBF_	0.0335	[0.0332, 0.0339]
	Male calf	μ_1XDM_	0.0392	[0.0389, 0.0396]	**μ**_1XBM_	0.0352	[0.0347, 0.0357]

**Figure 3:**
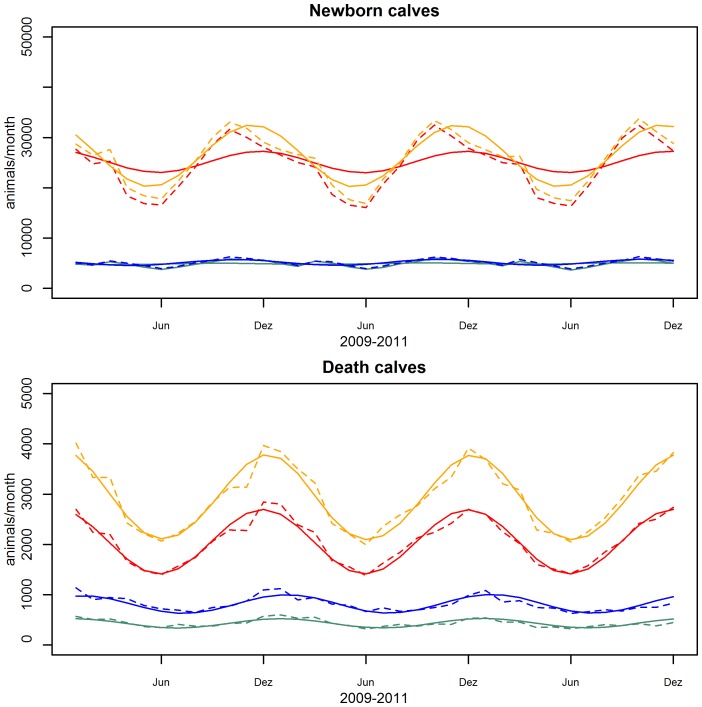
Seasonal pattern of birth and mortality in calves. Solid line: model data, dashed lines: AMD data. Orange: dairy male calf, red: dairy female calf, blue: beef male calf, green: beef female calf.

D: dairy; B: beef; F: female; M: male; X: calf, Y: subadult, Z: adult. Small letters indicate rates (s: slaughter, m: mortality, f: fattening, tr: transition to next age class). μ1: average birth rate; μ2: average mortality rate;

By introducing parameters (amplitude and phase, table 5) to describe calf mortality and birth rates as trigonometric functions, the seasonal dynamic of changes in the population can be described more accurately than with the corresponding linear parameters deducted from the monthly extracts of the AMD (figure 3).

**Table 5:** Values for the amplitudes and phases in the trigonometric functions of the presented Swiss cattle population model.

	Dairy			Beef	
		95%-CI			95%-CI
**a**_1XDF_	0.0031	[0.0022, 0.0041]	**a**_1XBF_	0.0009	[0, 0.0023]
**a**_1XDM_	0.0091	[0.0073, 0.0109]	**a**_1XBM_	0.0040	[0.0024, 0.0056]
**a**_2XDF_	0.0029	[0.0020, 0.0038]	**a**_2XBF_	0.0013	[0.0008, 0.0018]
**a**_2XDM_	0.0063	[0.0037, 0.0088]	**a**_2XBM_	0.0016	[0.0010, 0.0022]
**φ**_1XDF_	1.6799	[1.4046, 1.9574]	**φ**_1XBF_	2.9510	[1.1437, 4.7768]
**φ**_1XDM_	1.9245	[1.7428, 2.1096]	**φ**_1XBM_	2.4772	[2.0699, 2.8935]
**φ**_2XDF_	1.6576	[1.3443, 1.9727]	**φ**_2XBF_	1.0713	[0.6834, 1.4582]
**φ**_2XDM_	1.7900	[1.3820, 2.1969]	**φ**_2XBM_	0.9218	[0.5575, 1.2856]

**Figure 4:**
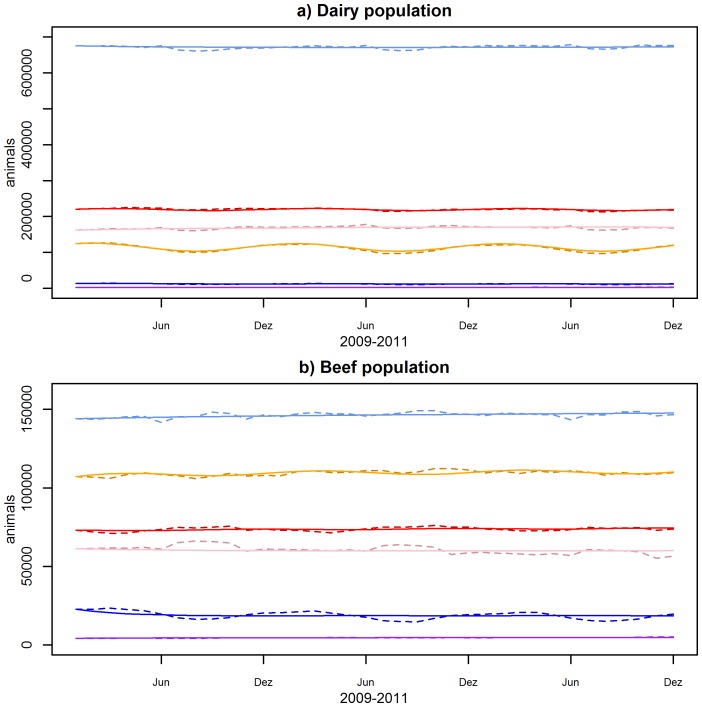
Animal numbers per age category. a) Dairy population. b) Beef population. Solid line: model data, dashed lines: AMD data. Light blue: cow, orange: male calf, red: female calf, pink: heifer, blue: young bull, purple: bull.

D: dairy; B: beef; F: female; M: male; X: calf, Y: subadult, Z: adult. a 1: amplitude for birth rate; a 2: amplitude for mortality rate; φ_1_: phase for birth rate; φ_2_: phase for mortality rate;

The correlation of the empirical parameters from the AMD and the fitted values gives a correlation coefficient of 0.994. The good fit of the model to the empirical data is also illustrated in figure 4.

As expected, the beef and dairy sector show differences in the demographic composition. While the proportions of young female animals are comparable (18.5% dairy female calves, 17.8% beef female calves 14.2% dairy heifers and 14.6% beef heifers), dairy cows account for around 56.7% of the dairy population while beef cows account for 35.5% of the beef population. For male animals the differences are even more noticeable: beef male calves, young bulls and bulls make 26.5%, 4.6% and 1.1% of the beef population compared to 9.6%, 1.0% and 0.2% for dairy male calves, dairy young bulls and dairy bulls respectively (all proportions are means over the 36 month of data analysis).

**Figure 5:**
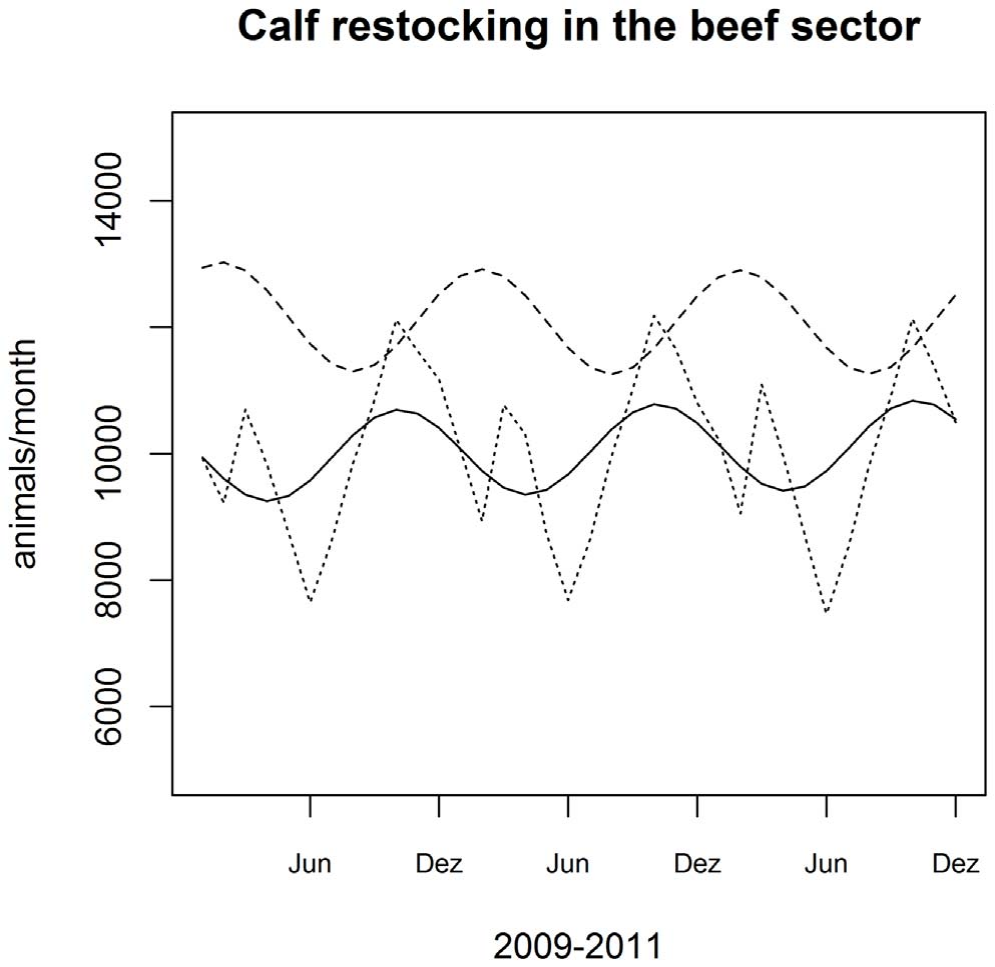
Restocking of calves in the beef sector. Dashed line: dairy calves transferred to fattening plants (VENSIM), solid line:born beef calves (VENSIM), dotted line: born beef calves (AMD).

As import and export of live cattle are negligible for Switzerland (6′787 imported animals from 2009 to 2011 and 3′318 exported animals over the same period), the beef population is maintained to a considerable extend by calves from the dairy industry. Almost every month more dairy calves are transferred to fattening plants (i.e. to from the dairy to the beef industry) than were born within the beef industry (figure 5).

**Figure 6:**
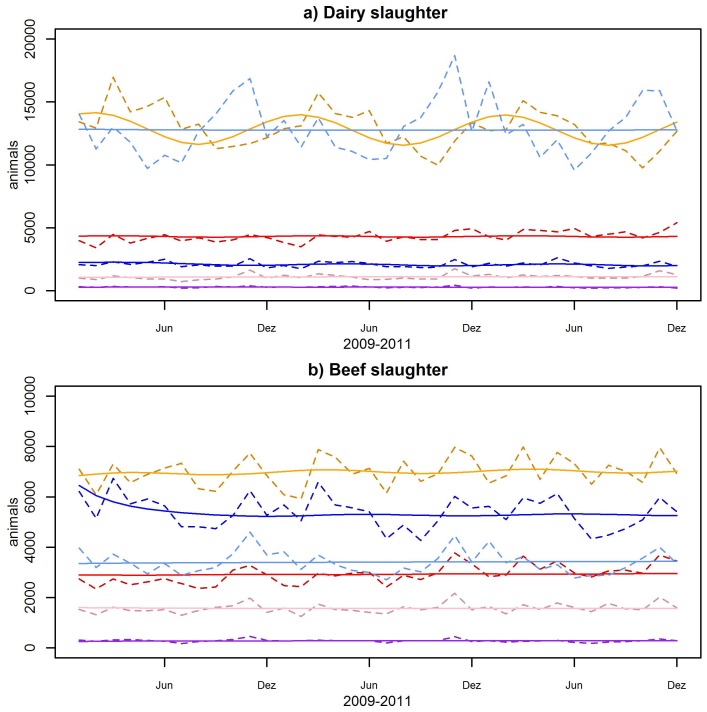
Slaughter numbers per age category. a) Dairy population. b) Beef population. Solid line: model data, dashed lines: AMD data. Light blue: cow, orange: male calf, red: female calf, pink: heifer, blue: young bull, purple: bull.

The number of slaughtered animals does not show a clear seasonal pattern (AMD data, figure 6) and the slaughter rate in the model is linear.

The sensitivity analysis shows, that the dairy female calf birth average and the dairy cow slaughter rate have the biggest influence on the total population with a change in animal numbers bigger than 50′000 after 3 years of simulation (figures S1–S10, supplementary material).

## Discussion

### 4.1 The Swiss cattle population

The composition of the Swiss cattle population accentuates that the milk industry dominates the domestic production and shapes the population dynamic. Adult dairy cows account for over 40% of all animals (figure 4). The importance of dairy female animals for the total population is reflected in the high sensitivity of the beef population to changes in the dairy cow slaughter rate and the dairy female birth rate (figures S2, S6, supplementary material). The irregular slaughter pattern indicates that the farmers keep the population constant by management decisions.

The higher monthly average mortality of dairy male calves compared to their contemporaries (0.0255 compared to 0.0094 (XDF), 0.0074 (XBM) and 0.0059 (XBF)) is in line with findings of other authors. [14] and [15] found higher mortality rates in dairy breeds than in beef breeds and higher mortality rates in male calves than female calves. As they all defined calves as maximum 180 days of age, the broader categories in our model might explain why dairy male calves differ as much from the others as the effect of early perinatal mortality with higher risk of dystocia for male calves [14] is combined with management decisions, i.e. less care for the economically relatively uninteresting male dairy calves [15]. As we also determined the production type on farm level and not according to the breed as in the above mentioned studies, effects of management decisions on the calve mortality might be even more manifest.

When deducting yearly rates roughly by multiplying the monthly age transition rates by 12, the difference in the management of beef and dairy animals becomes more obvious: while 82% of female dairy calves reach the next age class, only 25% of dairy male calves live through their first year. For beef calves 86% of the females and 61% of the males reach the next age class which reflects the interest of fattening beef breeds for more than 12 month. The most valued group of animals, dairy heifers, reach adulthood in 96% of the cases while more beef heifers are slaughtered and only 74% get two years old.

### 4.2 Model assumptions

In high productive agriculture systems of the developed world the population dynamics of livestock is controlled by the farmer and depends on policy and economics rather than on resource limitation or other external factors e.g. [16]. Bleul [14] states, that 80% of Swiss cows are inseminated artificially. For this reason we did not consider a resource constraint i.e. a carrying capacity in our model. The results may be of use for countries in similar economic situation but with less complete records but are to be applied carefully to cattle population that live under more resource dependent natural conditions.

The difference in the birth rates of dairy female and male calves in the model is an artefact presumably due to the difference in the dynamic of the two compartments. Dairy female calves are the most important segment to maintain the population which makes the model sensitive to any change in dairy female calf births. A conservative simulation gives a more stable overall result.

As alpine pastures usually use the gained milk directly for cheese production and it enters therefore not in commerce or they have young stock not yet lactating, they are mostly in the beef category regardless the provenience of the cattle. Therefore the data was corrected over the summer months, using the production type of the farm of origin from the movement records to alpine pastures. The visible seasonal bumps in beef heifers in figure 4 show, that the correction is imperfect due to an incomplete registration of the movements from and to alpine pastures. Since 2012 these are mandatory and improvement of the data quality can be expected.

To integrate the seasonality of birth and mortality in calves, we assumed a sinusoidal pattern and did not investigate other functions.

### 4.3 Future applications of the model

This is the first dynamic population model for Swiss cattle. As the data source is the complete record of the cattle population, a very good fit could be expected. Nonetheless the fitted population parameters allow a close to reality simulation of the population for future development planning scenario analysis, serve as a backbone to disease transmission models and for the simulation of disease surveillance and control (e.g. [17]).

The fitted population parameters allow building age and sex structured transmission models to simulate disease dynamics with different prevalences in different age classes (e.g. infectious bovine rhinotracheitis IBR, Brucellosis).

Furthermore the transmission rates of different age and production type categories to the slaughterhouse give precise information, which proportions of populations and subpopulations would be basically available for testing at the slaughterhouse in which time period. The slaughterhouse is a very convenient spot for sampling, because it allows taking samples from many animals from different farms of origin within a short time period. Furthermore, there are diseases such as bovine spongiform encephalopathy (BSE) that can only be diagnosed in tissue matrices accessible at slaughter, e.g. brainstem.

As the outcome parameters in the model are calculated for the dairy and beef sector separately, surveillance systems with different components for the different production types can be simulated (e.g. IBR, Brucellosis). For example the efficacy of combining bulk tank milk sampling with slaughterhouse or on farm sampling can be evaluated. As the transfer from calves from the dairy sector to the beef sector is included, the model allows a realistic simulation of disease transmission in the overall population and of the effect of different surveillance strategies on the system sensitivity for different production types.

The fitted population parameters can also be interpreted as baseline parameters for the healthy Swiss cattle population. As seasonal effects are included in the parameter fitting, they can be used to search for aberrations in present data (e.g. increased mortality) to detect health events in an early stage.

In the healthy population most female calves are kept to restock the dairy population, as can be inferred from the relatively low transmission rates of female dairy calves to slaughter. If that segment is affected by an epidemic leading to increased abortions, calf mortality or decreased fertility, consequences on population structure and management are to be expected. Achievement of breeding objectives might be delayed or even out of reach. Impacts on the milk and meat markets are to be expected. The impact on population structure such as decrease of adult dairy cows in the slaughter population can be estimated by model derived transmission factors.

## Conclusions

The Swiss animal movement database is a reliable source of information about the Swiss cattle population and can provide stakeholders and decision makers with important knowledge without expensive and laborious field work. The presented demographic model allows a simulation of Swiss cattle production and economics under different policy scenarios and can be used as the demographic backbone for disease transmission models.

## Supporting Information

Figure S1 **Influence of varying slaughter rates on the number of animals in the dairy population.**
(TIF)

Figure S2 **Influence of varying slaughter rates on the number of animals in the beef population.**
(TIF)

Figure S3 **Influence of varying mortality rates on the number of animals in the dairy population.**
(TIF)

Figure S4 **Influence of varying mortality rates on the number of animals in the beef population.**
(TIF)

Figure S5 **Influence of varying average birth rates on the number of animals in the dairy population.**
(TIF)

Figure S6 **Influence of varying average birth rates on the number of animals in the beef population.**
(TIF)

Figure S7 **Influence of varying fattening rates (calves transferring from the dairy to the beef sector) on the number of animals in the dairy population.**
(TIF)

Figure S8 **Influence of varying fattening rates (calves transferring from the dairy to the beef sector) on the number of animals in the beef population.**
(TIF)

Figure S9 **legends for the colour scales for the dairy population.**
(TIF)

Figure S10 **legends for the colour scales for the beef population.**
(TIF)

Text S1 (DOCX)
