# Supplementary figures and images for: Demographic Model of the Swiss Cattle Population for the Years 2009-2011 Stratified by Gender, Age and Production Type
Source: PLoS One. 2014 Oct 13;9(10):e109329. doi: 10.1371/journal.pone.0109329 (PMC4195665; doi:10.1371/journal.pone.0109329)

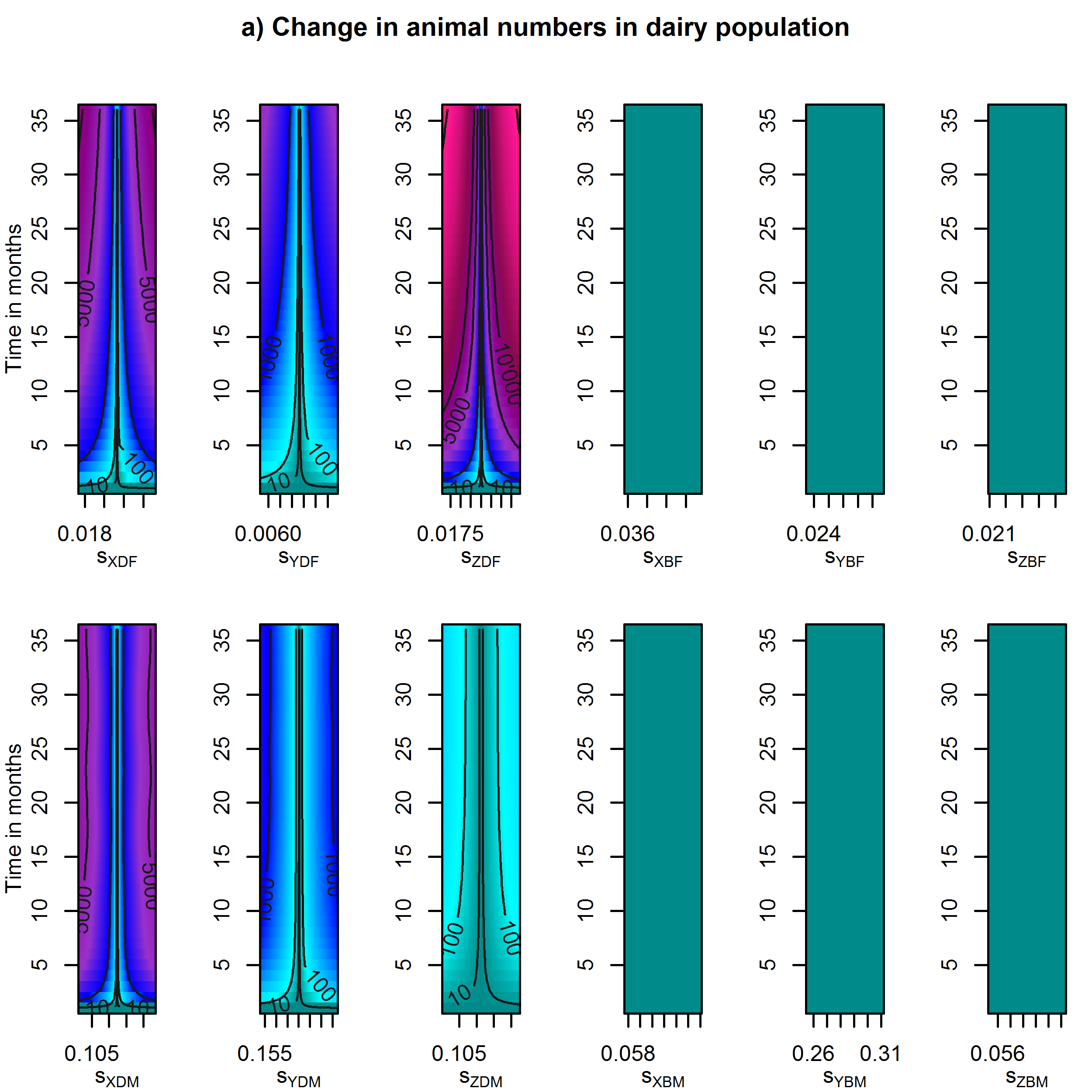

Supplement: Figure S1 — Influence of varying slaughter rates on the number of animals in the dairy population. (TIF) [file pone.0109329.s001.tif]

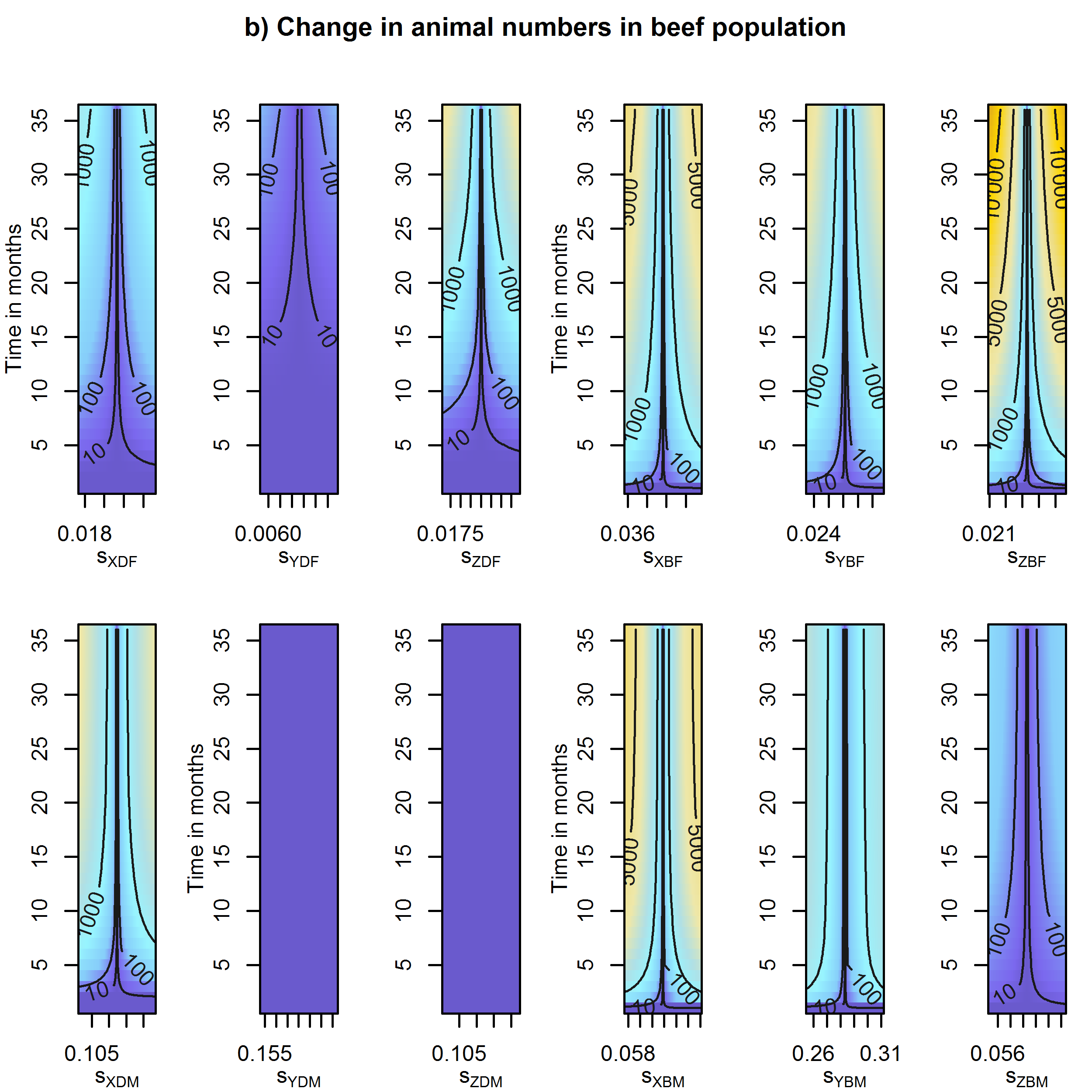

Supplement: Figure S2 — Influence of varying slaughter rates on the number of animals in the beef population. (TIF) [file pone.0109329.s002.tif]

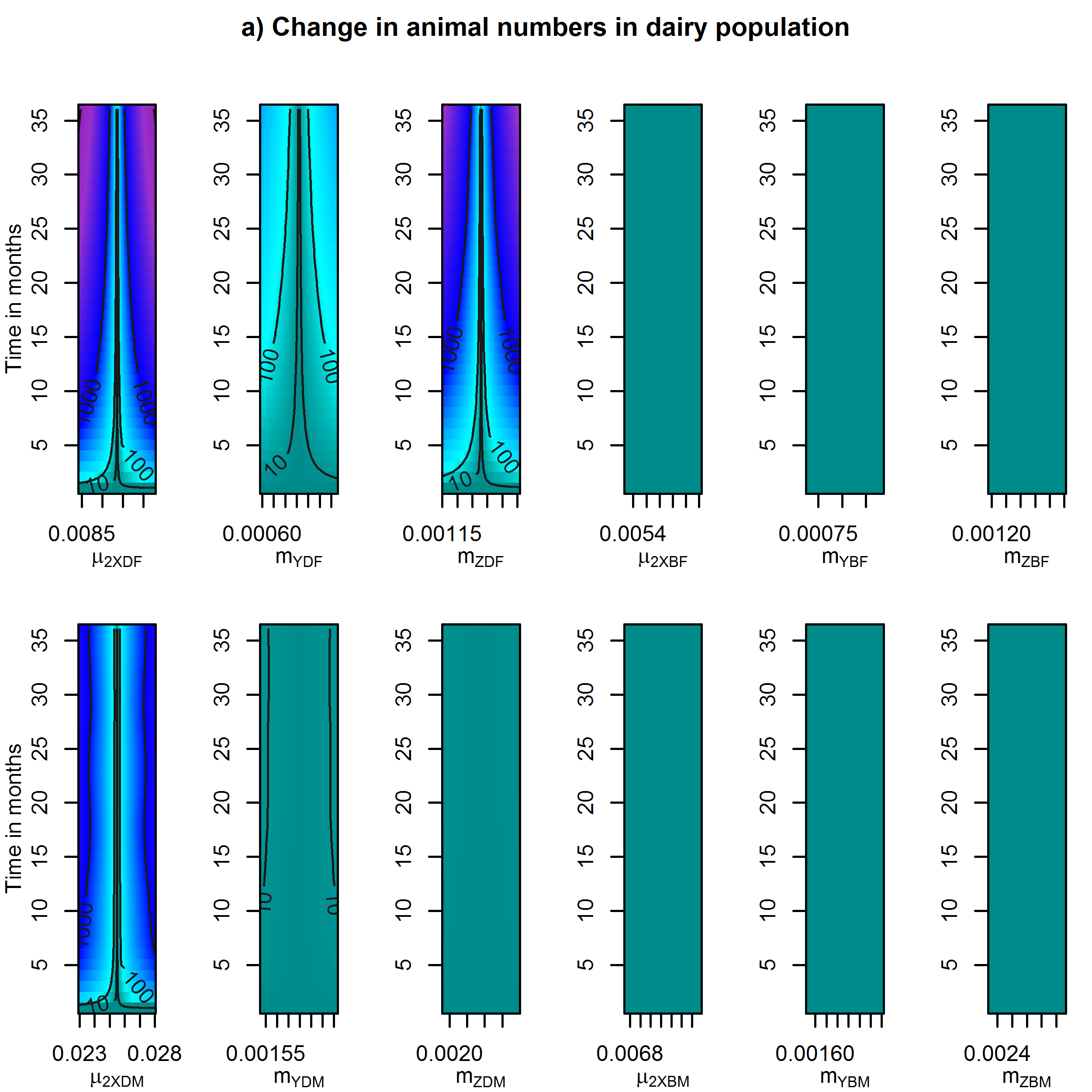

Supplement: Figure S3 — Influence of varying mortality rates on the number of animals in the dairy population. (TIF) [file pone.0109329.s003.tif]

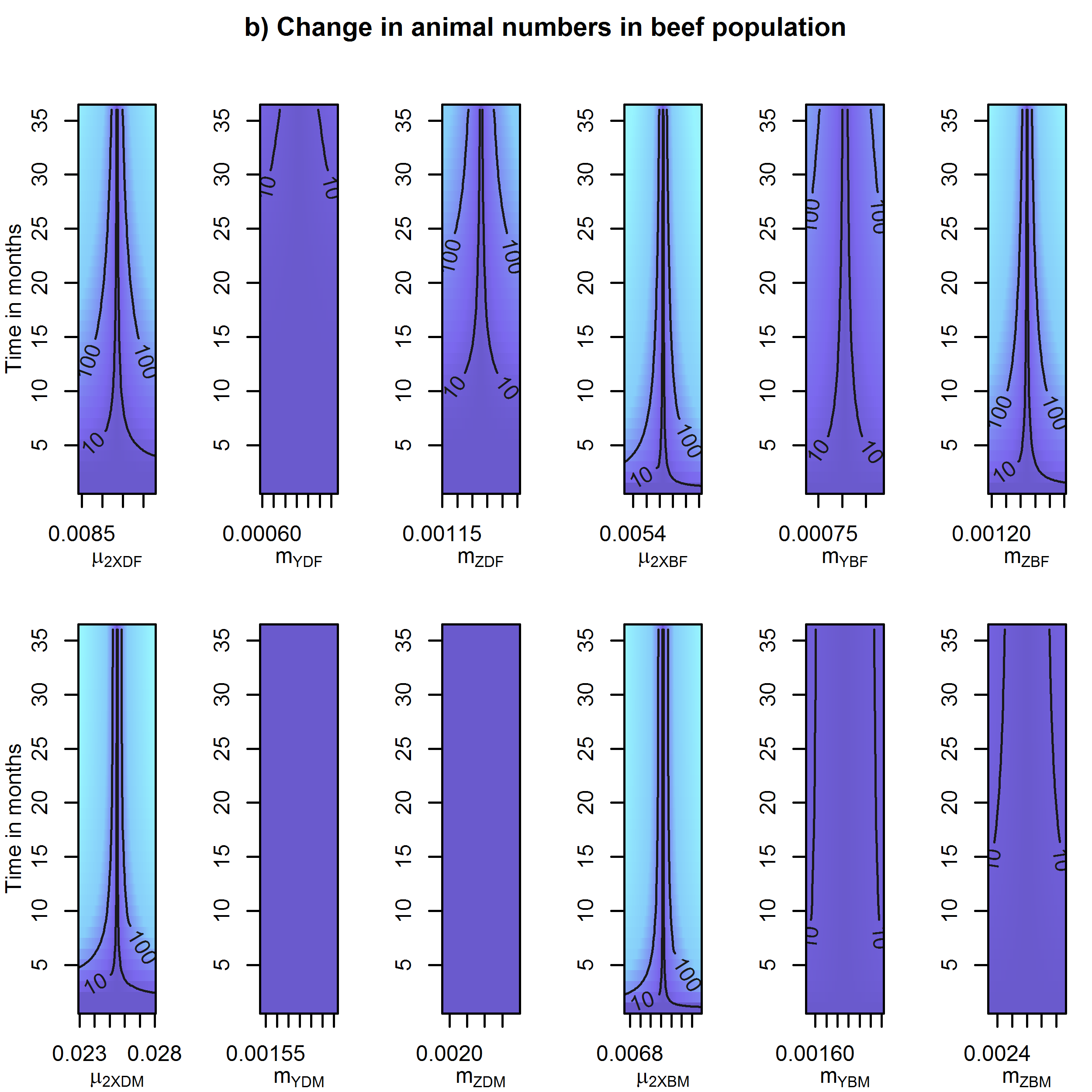

Supplement: Figure S4 — Influence of varying mortality rates on the number of animals in the beef population. (TIF) [file pone.0109329.s004.tif]

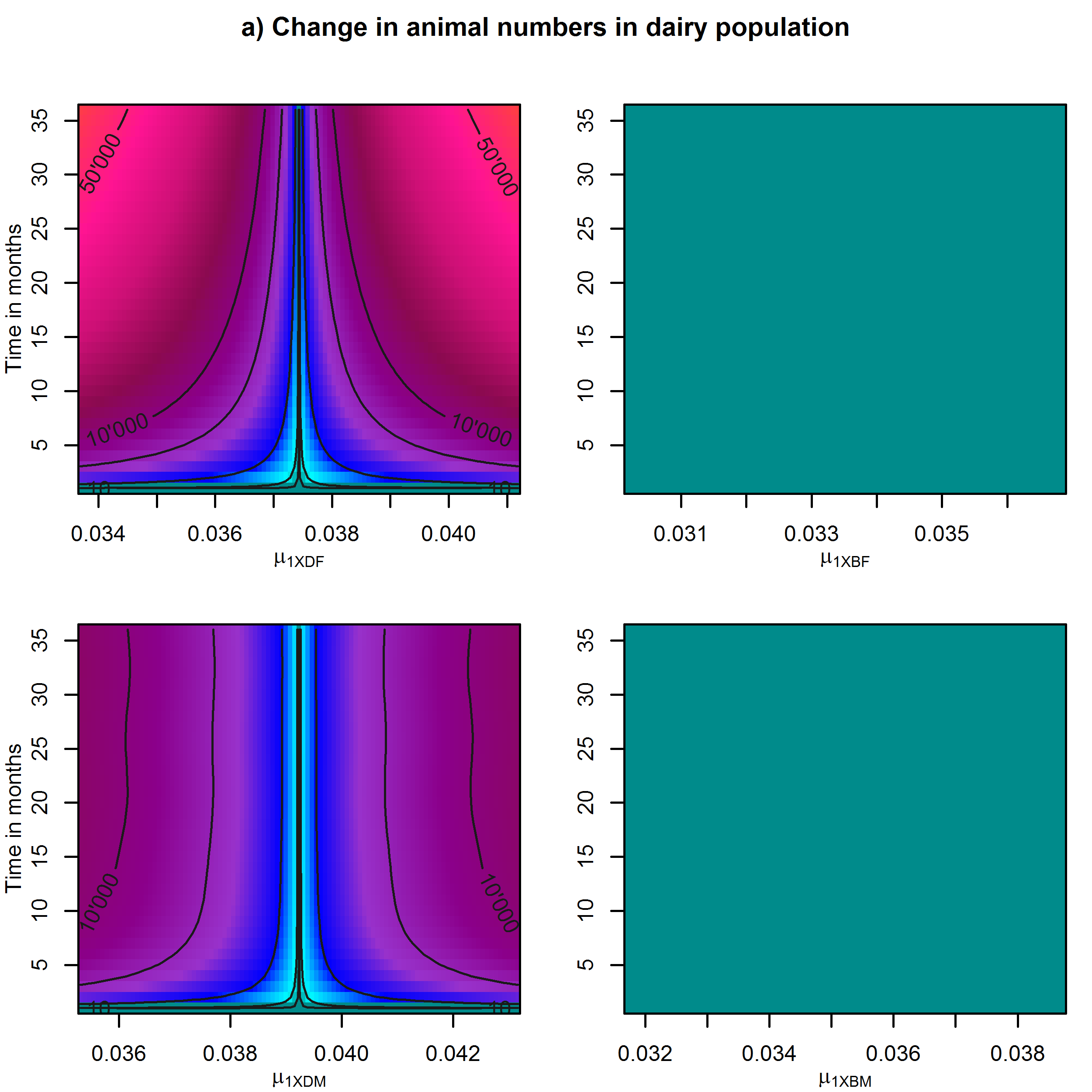

Supplement: Figure S5 — Influence of varying average birth rates on the number of animals in the dairy population. (TIF) [file pone.0109329.s005.tif]

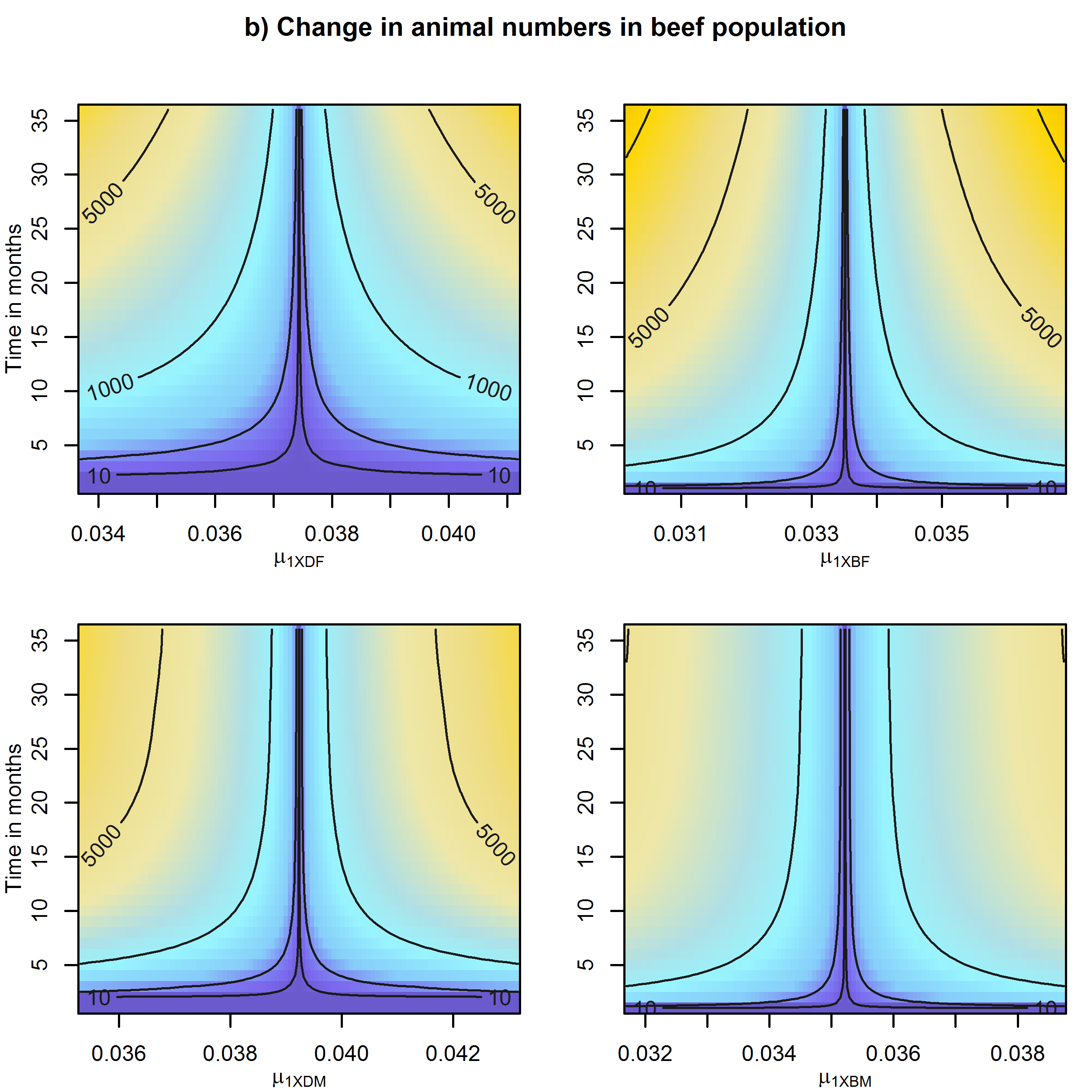

Supplement: Figure S6 — Influence of varying average birth rates on the number of animals in the beef population. (TIF) [file pone.0109329.s006.tif]

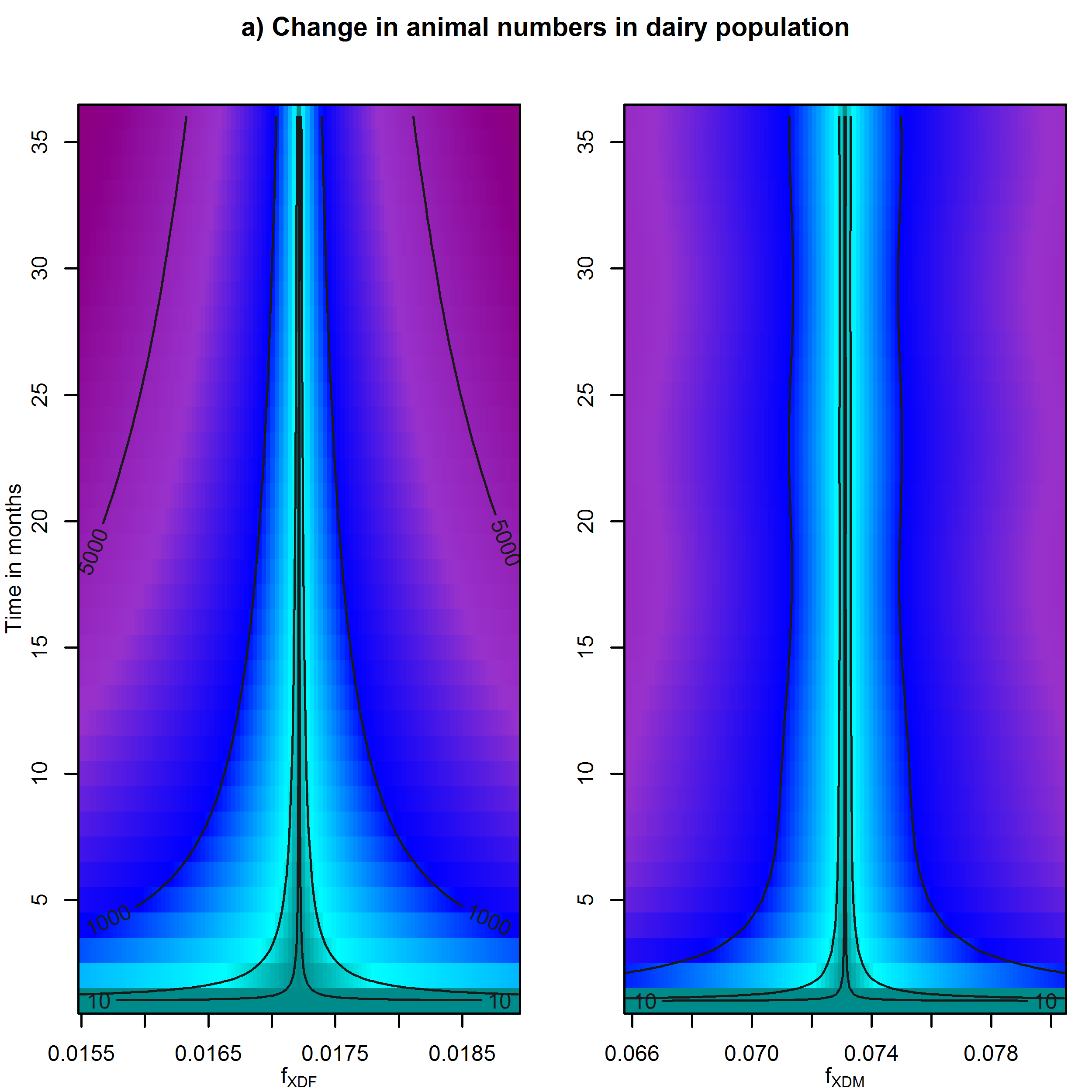

Supplement: Figure S7 — Influence of varying fattening rates (calves transferring from the dairy to the beef sector) on the number of animals in the dairy population. (TIF) [file pone.0109329.s007.tif]

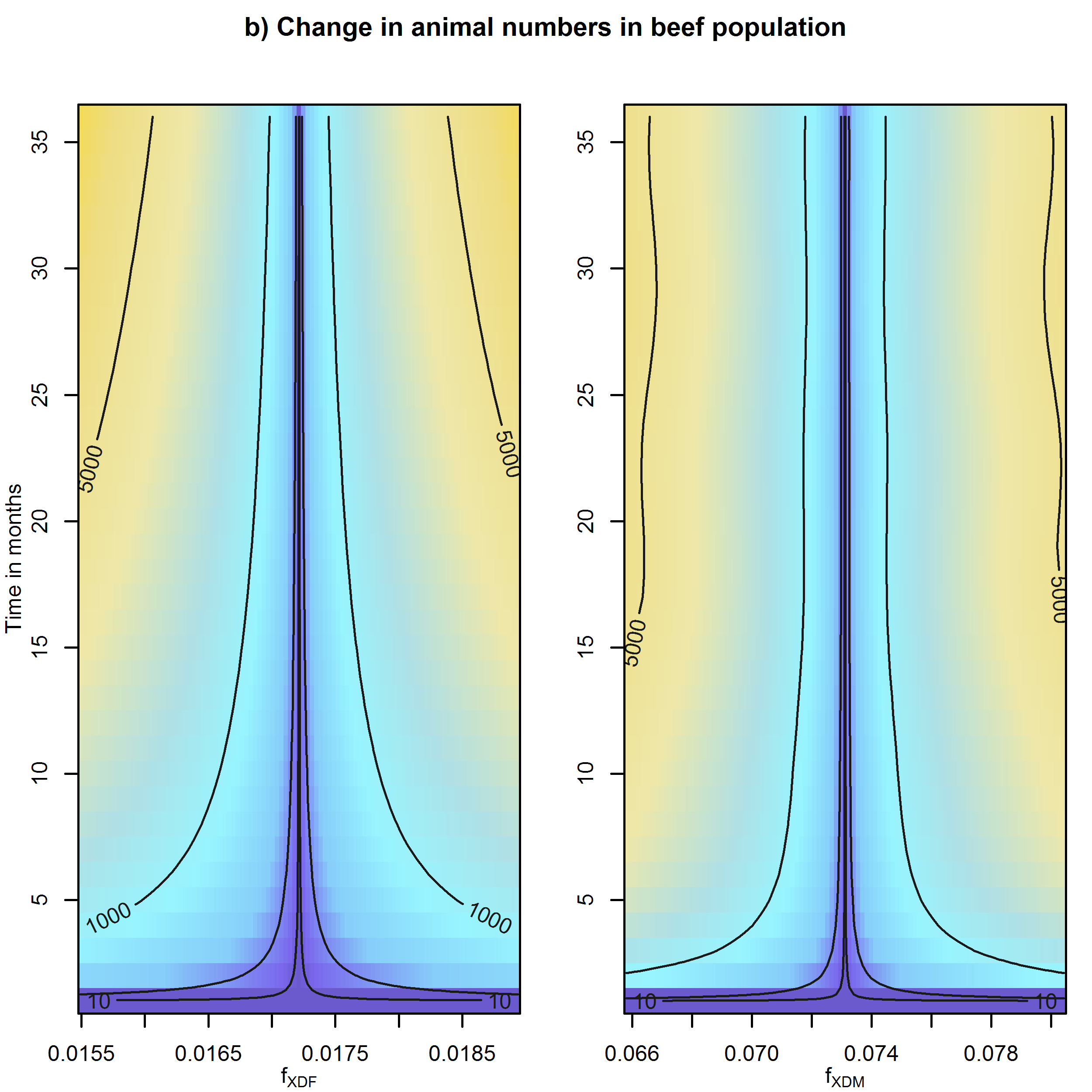

Supplement: Figure S8 — Influence of varying fattening rates (calves transferring from the dairy to the beef sector) on the number of animals in the beef population. (TIF) [file pone.0109329.s008.tif]

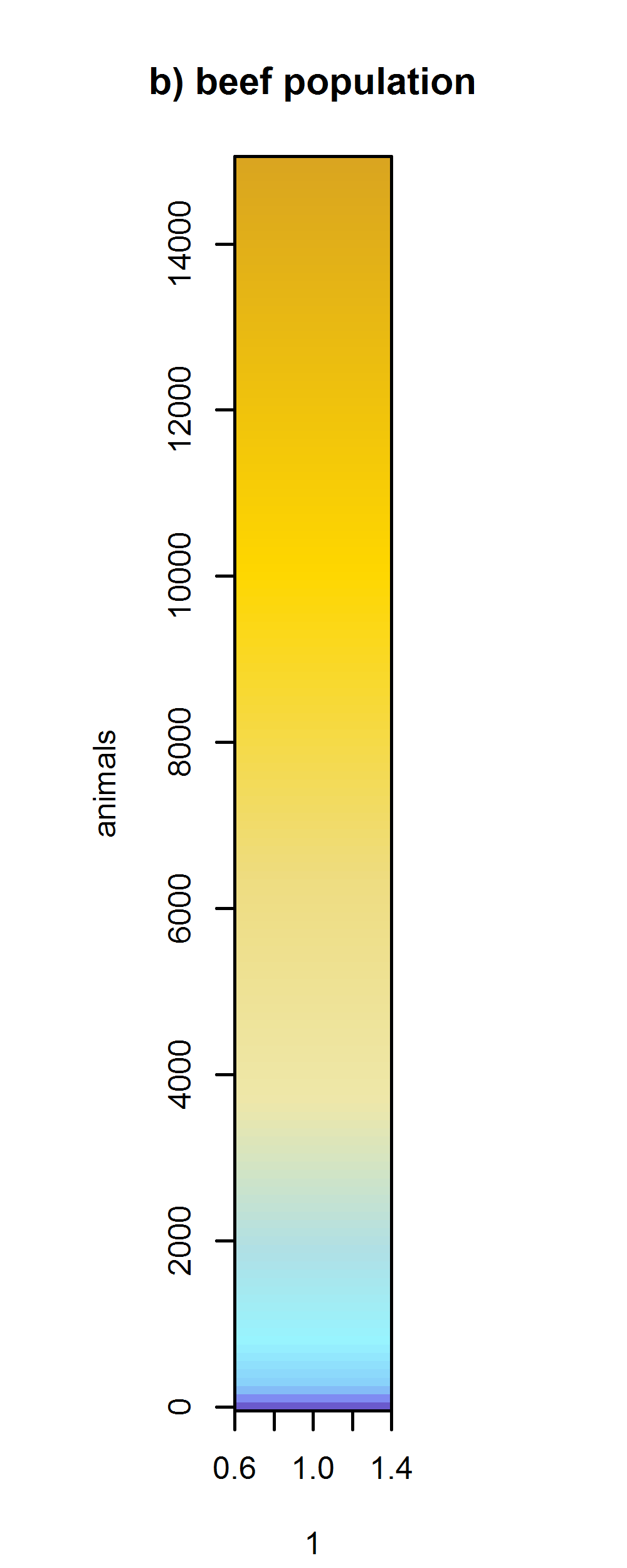

Supplement: Figure S9 — legends for the colour scales for the dairy population. (TIF) [file pone.0109329.s009.tif]

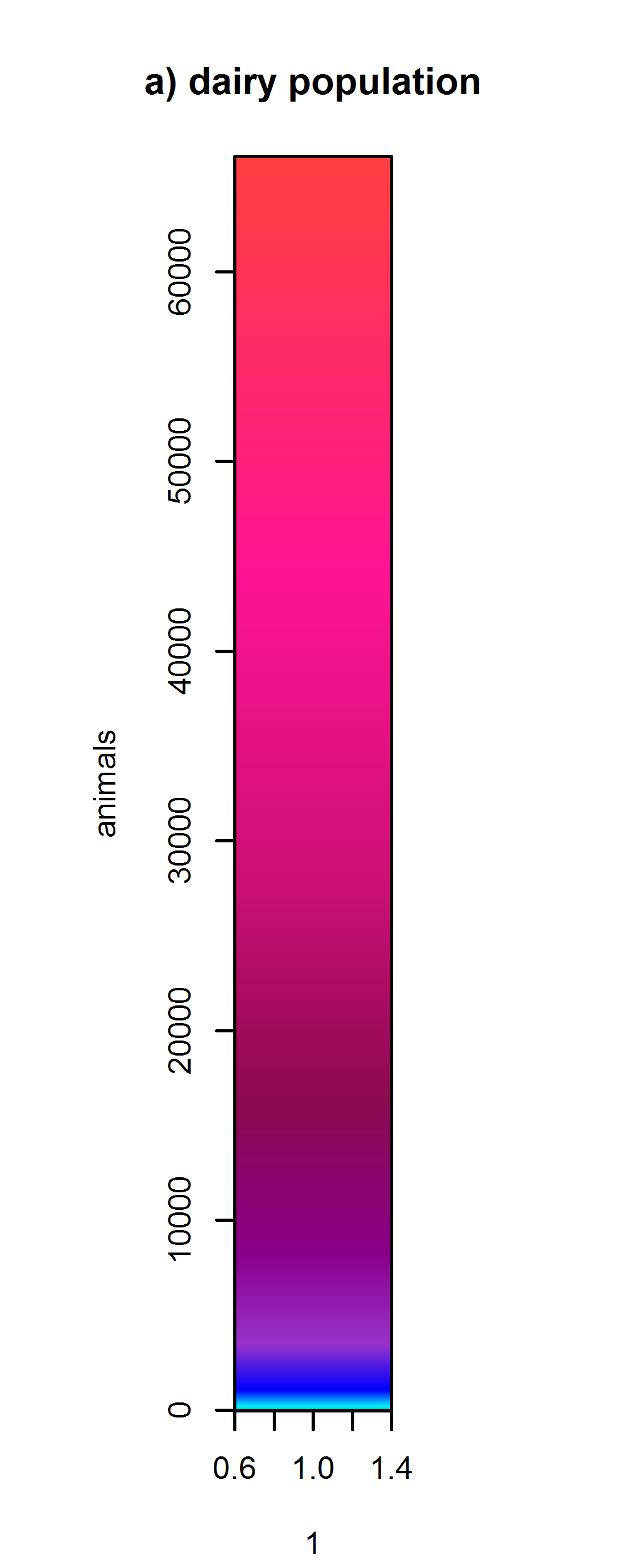

Supplement: Figure S10 — legends for the colour scales for the beef population. (TIF) [file pone.0109329.s010.tif]
