# Supplementary material for: Demographic Model of the Swiss Cattle Population for the Years 2009-2011 Stratified by Gender, Age and Production Type
Source: PLoS One. 2014 Oct 13;9(10):e109329. doi: 10.1371/journal.pone.0109329 (PMC4195665; doi:10.1371/journal.pone.0109329)
Supplement: Text S1 — (DOCX) [file pone.0109329.s011.docx]

**Text S1: Sensitivity analysis**

The graphical representation of the sensitivity analysis of the demographic model of the Swiss cattle population shows, how the parameters in the model influence the number of animals in the total, the dairy and the beef population respectively. Each parameter was varied separately using a range from -10% to +10% of the fitted value from the Vensim model (baseline), divided in 100 steps.

The figures S1-S4 show the change in numbers of animals using different colour palettes for the change in the dairy population (aquamarine-blue-magenta) and the beef population (violet-turquoise-yellow). Contour lines indicate the absolute change in number of animals (10, 100, 1’000, 5’000, 10'000, 50'000).
